# Influenza a virus-triggered autophagy decreases the pluripotency of human-induced pluripotent stem cells

**DOI:** 10.1038/s41419-019-1567-4

**Published:** 2019-04-18

**Authors:** Ali Zahedi-Amiri, Glen L. Sequiera, Sanjiv Dhingra, Kevin M. Coombs

**Affiliations:** 1 Department of Medical Microbiology and Infectious Diseases, University of Manitoba, Winnipeg, MB Canada; 2 Manitoba Centre for Proteomics and Systems Biology, Winnipeg, MB Canada; 3 Department of Physiology and Pathophysiology, University of Manitoba, Winnipeg, MB Canada; 4 Institute of Cardiovascular Sciences, Albrechtsen Research Centre, St. Boniface Hospital, Winnipeg, MB Canada; 5 Children’s Hospital Research Institute of Manitoba, Winnipeg, MB Canada

**Keywords:** Protein-protein interaction networks, Macroautophagy, Pluripotency, Induced pluripotent stem cells, Infection

## Abstract

Maternal influenza infection during pregnancy was reported multiple times as the possible cause of many defects and congenital anomalies. Apart from several cases of influenza-related miscarriage during various trimesters of pregnancy, some epidemiological data suggest a link between maternal influenza infection and genetic abnormalities in offspring. However, there are no reports yet describing how maternal influenza alters cellular pathways at early stages of development to result in congenital defects in the fetus. In the present study, using proteomic approaches, we utilized human-induced pluripotent stem cells (hiPSCs) for modeling intrablastocyst infection with influenza virus to not only investigate the vulnerability and responses of pluripotent stem cells to this virus but also to determine the possible impacts of influenza on pluripotency and signaling pathways controlling differentiation and embryogenesis. Our data indicated viral protein production in influenza A virus (IAV)-infected hiPSCs. However, viral replication was restricted in these cells, but cell viability and pluripotency were negatively affected. These events occurred simultaneously with an excessive level of IAV-induced autophagy as well as cytopathic effects. Quantitative SOMAscan screening also indicated that changes in the proteome of hiPSCs corresponded to abnormal differentiation in these cells. Taken together, our results showed that IAV-modulated reduction in hiPSC pluripotency is associated with significant activation of autophagy. Further investigations are required to explore the role of IAV-induced autophagy in leading pluripotent stem cells toward abnormal differentiation and impaired development in early stages of embryogenesis.

## Introduction

Some influenza pandemics and seasonal epidemics have shown high rates of fatalities in pregnant women and the fetus, suggesting that pregnancy puts both mother and offspring at higher risk of developing flu complications^1^. In addition to influenza-induced miscarriages, maternal influenza infection was reported several times as possible cause of some developmental malformations, but the molecular mechanisms behind such teratogenic effects have remained elusive^2–6^. A few pathologic analyses have not only confirmed viremia and extrapulmonary spread of influenza in pregnant women but also its transmission to fetal tissues^7–10^. Influenza-modulated congenital defects are mostly believed to originate from early pregnancy when the unevolved and vulnerable placenta still develops. Although transplacental passage of this virus is debated, embryonic cells can be affected through feto-maternal viral interference in signaling pathways controlling embryogenesis. Influenza replicates in certain multipotent stem cells^11–13^. Nevertheless, Influenza A virus (IAV) has limited replication in mouse embryonic stem cells (mESCs), but reduces their viability even at low multiplicity of infection (MOI), indicating the vulnerability of the blastocyst’s inner cell mass (ICM) to IAV^14^. ESCs are pluripotent and present only transiently at the ICM before initiating embryogenesis. Pluripotency is an essential attribute of pluripotent stem cells (PSCs) and represents the stemness, differentiation capacity, and self-renewal ability of such cells. Interactions of influenza proteins with themselves and with host cell proteins elicit substantial changes in the proteome to expand viral replication, as noticed by several virus-induced impairments in host cellular machinery affecting apoptosis and autophagy, immune responses, and protein synthesis^15–21^. None of these influenza-mediated alterations have been studied under the pluripotent state on a proteomic scale. Cell death and survival signaling pathways in these, especially after viral infections, are also poorly understood^22^. Additionally, modifications in pluripotency, self-renewal, and differentiation, as the most critical characteristics of PSCs, have not yet been elucidated under infections with most viruses, including influenza. Human-induced pluripotent stem cells (hiPSCs), which are generated through reprograming somatic cells, show great promise as both ethical and functional alternatives to ESCs for in vitro modeling of intrablastocyst infections. In the present study, we investigated the effects of IAV on the pluripotency and the proteome of hiPSCs.

## Results

### Influenza A/PR/8/34 replicates restrictively in hiPSCs

**Fig. 1:**
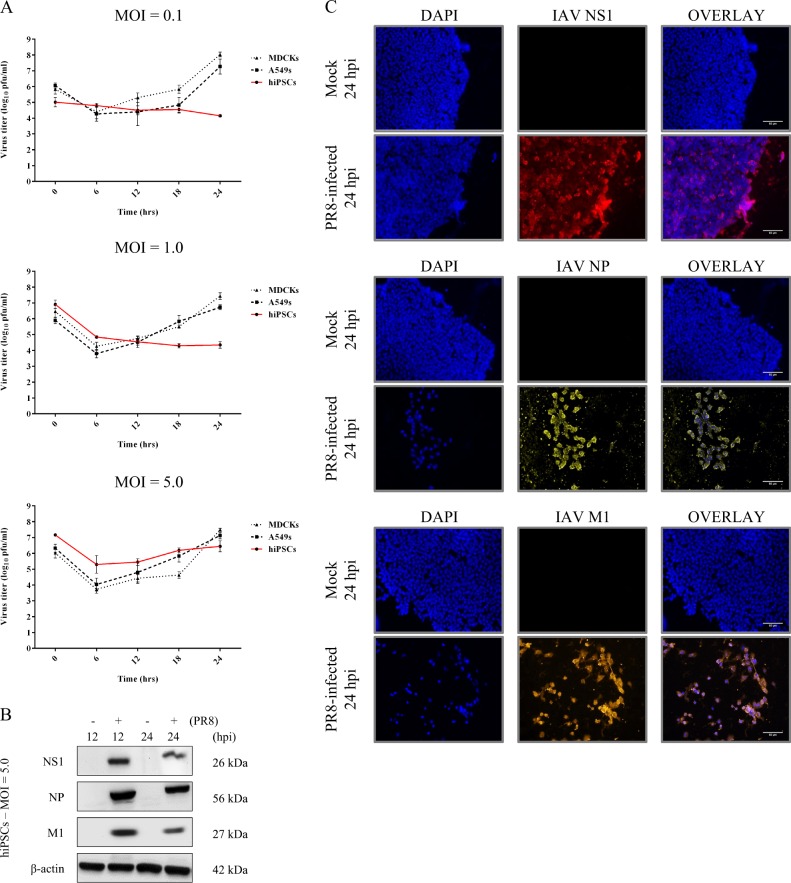
Restricted replication of influenza A/PR/8/34 in hiPSCs. **a** Comparison of PR8 virus growth kinetics between hiPSCs and IAV-permissive epithelial cell lines. hiPS, MDCK, and A549 cells were infected at various MOIs to track and compare viral yield. Supernatants were collected and titered in MDCK cells to measure viral growth by the standard plaque assay at different time points. **b** Immunoblot detection of IAV protein production in pluripotent stem cells. hiPSCs were infected with influenza A/PR/8/34 at MOI = 5 PFU/cell, and protein lysates were examined for production of viral NS1, NP, and M1 proteins at 12 and 24 h post infection by immunoblotting. **c** Validation of PR8 protein production in iPS cells by immunofluorescent microscopy. hiPS cells were infected for 24 h at MOI = 5 PFU/cell. After fixation, cells were probed with primary antibodies for viral proteins and imaged with a fluorescence microscope to detect NP (yellow), NS1 (red), and M1 (orange). Nuclei = blue (scale bar = 50 μm)

Unlike MDCK and A549 epithelial cells, which are permissive to influenza, IAV growth was restricted in hiPSCs at MOIs of 0.1 and 1.0 (Fig. 1a, upper and middle panels). At 24 hpi, the maximum titer of 10^6^ PFU/mL was attained at MOI of 5.0, which appeared to be upward and a little lower than the initial titer at 0 hpi (Fig. 1a, bottom panel). Despite no detectable protein expression at lower MOIs, viral matrix 1 protein (M1), nucleoprotein (NP), and nonstructural-1 protein (NS1) were produced in hiPSCs by 12 hpi at MOI of 5 (Fig. 1b). We further validated the expression of viral proteins and also noticed that an MOI of 5 was adequate to establish infection in nearly 80% of cells by 24 hpi (Fig. 1c). Although the assembly and maturation of infectious progeny viruses appeared to be limited, these data collectively indicate that influenza A/PR/8/34 enters the nucleus of hiPSC at higher MOIs and can initiate transcription and replication.

### Influenza causes cytopathology and activates autophagy in hiPSCs

**Fig. 2:**
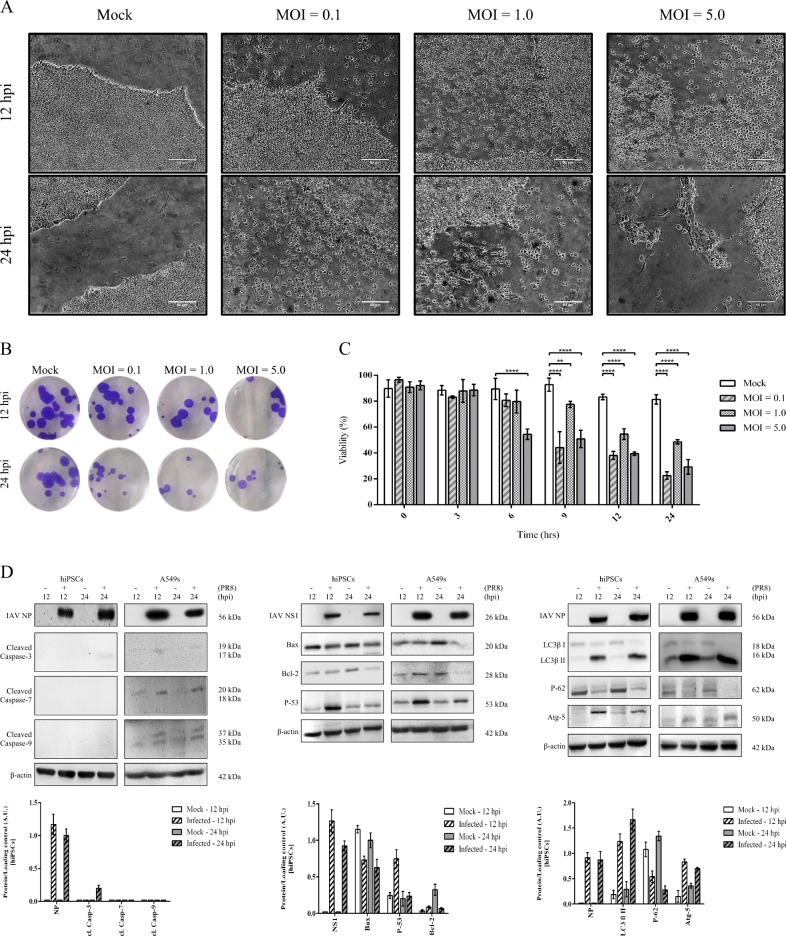
Influenza causes cytopathology and activates autophagy in hiPSCs. **a** Visual examination of IAV-induced CPE in pluripotent stem cells. Mock and virus-infected cells at different MOIs were observed under an inverted microscope and photographed at 12 and 24 hpi (scale bar= 50 μm). **b** Impact of IAV on the size and numbers of hiPSC colonies. hiPSC colonies were infected with PR8 at three MOIs for 12 and 24 h, then fixed with 4% paraformaldehyde, and stained with crystal violet. Mock-infected colonies of hiPSCs showed deeper staining than infected colonies due to higher cell density per colony. **c** Quantification of Trypan blue exclusion test of cell viability at different MOIs and postinfection time points. In contrast to mock-infected cells, which remained more than 80% viable for a day, the viability of PR8-infected cells reduced significantly at 24 hpi for all MOIs compared to the mock 0 time and time-matched mock controls (*P*-value ≤ 0.01 = **, *P*-value ≤ 0.0001 = ****). **d** Effect of IAV infection on intrinsic apoptosis and autophagy activation. Following infection with PR8 virus at MOI of 5, cell lysates were extracted at 12 and 24 hpi, then fractionated by SDS-PAGE and assessed by western blotting. IAV proteins NP, M1, and NS1 were used as markers of infection. Left panel: compared to A549 cells, PR8 infection did not elevate the cleavage of caspases-7, -3, -9, the main executioners of the intrinsic apoptotic pathway, in hiPSCs by 24 hpi. Middle panel: in contrast to PR8-infected A549 cells which showed clear decrease in the expression of Bax (proapoptotic) at 24 hpi, hiPSCs expressed relatively unchanged levels of this protein within 24 h of infection with influenza, despite the slight reduction in infected cells. The abundance of Bcl-2 (antiapoptotic) was severely decreased one day after infection in both hiPS and A549 cells. Infecting these pluripotent stem cells with PR8 virus resulted in transient p53 accumulation only at 12 hpi without subsequent production at the later time points. Right panel: similar to A549s, IAV-induced autophagy was detected based on LC3β-I to II conversion, Atg5 accumulation, and p62 degradation

By increasing the MOI, infected cells displayed more cytopathic effects (CPEs), such as shrinkage, rounding, detaching from the culture vessels, and notable reduction in the number and size of colonies, suggesting the induction of cell death (Fig. 2a, b). The viability of PR8-infected cells also was reduced (Fig. 2c). These observations confirm cytopathogenic entry of virus. Compared to A549 cells, IAV did not elevate the cleavage of caspases in hiPSCs by 24 hpi and showed unique cell-specific differences in the expression of apoptosis regulators like Bax, Bcl-2, and p53 (Fig. 2d, left and middle panels), highlighting constrained intrinsic apoptosis in hiPSCs after IAV infection. However, the induction of autophagy after influenza infection was demonstrated by detection of LC3β-II, Atg5, and p62 (Fig. 2d, right panel). Taken together, unlike limited intrinsic apoptosis, IAV activated autophagy in hiPSCs earlier than apoptosis executioners and to much higher levels.

### IAV infection diminishes pluripotency of hiPSCs

**Fig. 3:**
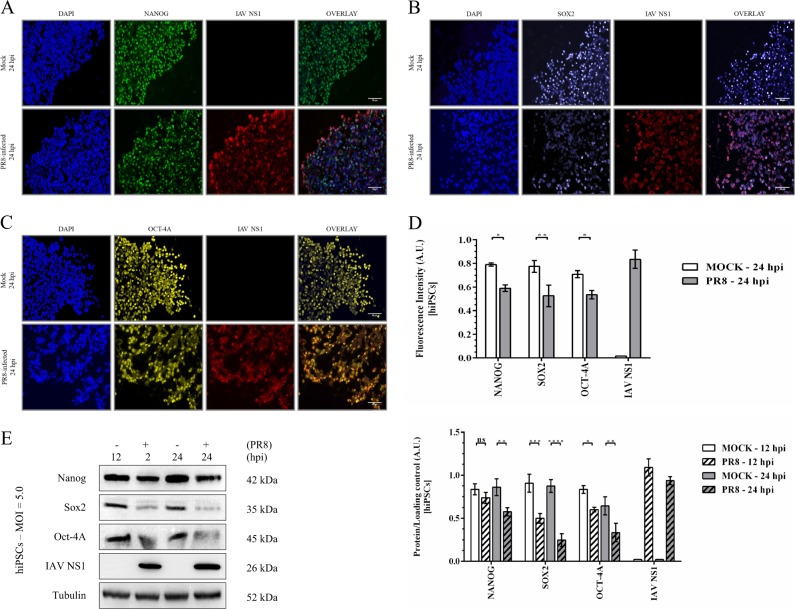
IAV is capable of reducing pluripotency. The expression levels of pluripotency markers Nanog (green) (**a**), Sox2 (indigo) (**b**), and Oct-4A (yellow) (**c**) were measured by immunofluorescence microscopy at 24 hpi in the presence of viral NS1 protein as a marker for productive IAV infection. DNA counterstain is shown in blue (scale bar = 50 μm). **d** Quantification of florescence intensity for pluripotency-regulating proteins as well as IAV NS1. Signals of detected proteins were normalized to the level of nuclear staining hiPSCs. **e** Western blot analysis validates the influenza-mediated loss of pluripotency in hiPSCs. *P*-value > 0.05 = ns, *P*-value ≤ 0.05 = *, *P*-value ≤ 0.01 = **, *P*-value ≤ 0.001 = ***, *P*-value ≤ 0.0001 = ****

Although most infected cells showed pluripotency characteristics by 12 hpi (data not shown), an overall decrease of at least 20% was observed in the expression of pluripotency regulating proteins Nanog, Sox2, and Oct-4A after 24 h at MOI of 5.0 (Fig. 3d). The validation of these results showed similar or greater reduction in levels of pluripotency indicators at 24 hpi (Fig. 3e). IAV NS1 was almost equal between cells producing less Sox2 (Fig. 3b) or Oct-4A (Fig. 3c) and cells with a considerable level of these two proteins. However, apart from edges of infected colonies, NS1 appeared to be more abundant in cells expressing diminished levels of Nanog (Fig. 3a), showing that PR8 virus decreases the pluripotency for expanding its infectivity in hiPSCs.

### Inhibition of autophagy maintains pluripotency and limits viral growth

**Fig. 4:**
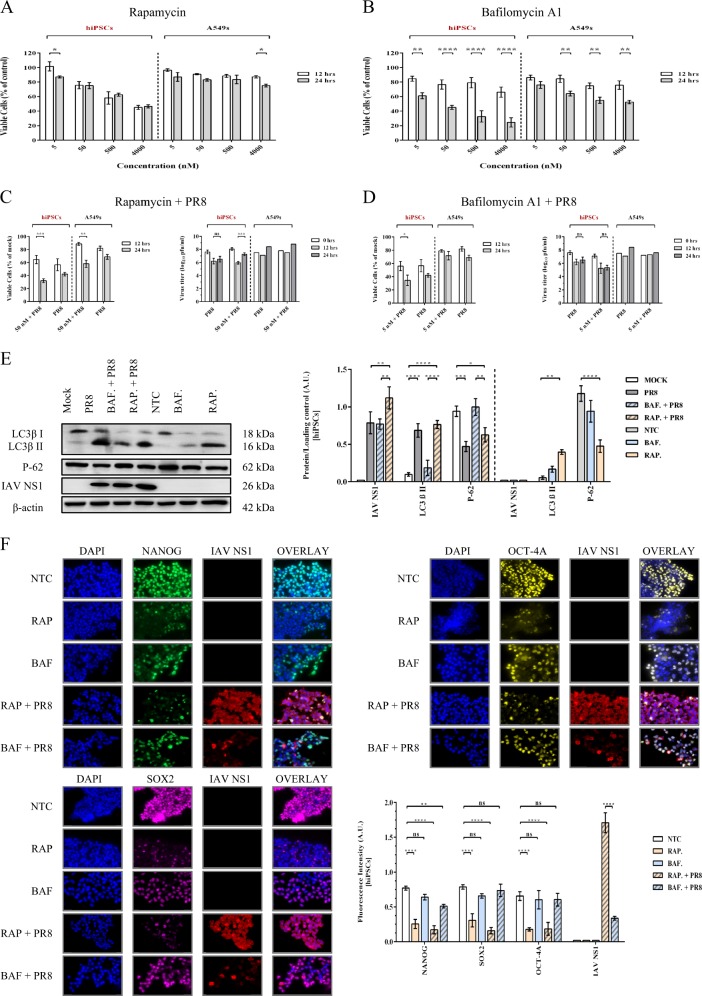
Effects of autophagy inhibition and induction on pluripotency and viral growth. Cytotoxicity of Rapamycin (**a**) and Bafilomycin A1 (**b**) in hiPSCs and A549s. After treating cells with various concentrations of these drugs, cell viability was assessed by the WST-1 cell proliferation assay at 12 and 24 h. **c** Rapamycin increases the influenza titer (right panel), despite reducing cell viability in presence of PR8 virus (left panel). **d** Significant decline of viral titer in infected Bafilomycin-treated cells (right panel) is also associated with reduced cell viability (left panel). Assessment of viral titer was done by the plaque assay. **e** Expression levels of autophagy markers after exposure to autophagy inducer and inhibitor in infected and noninfected hiPSCs. After treating with drugs, cell lysates were assayed by immunoblotting for detection of LC3β-I to II conversion and p62 degradation. **f** Induction of autophagy negatively affects pluripotency, while the inhibition of autophagic activity maintains this characteristic of pluripotent stem cells. The expression of pluripotency proteins Nanog (green), Sox2 (violet), and Oct-4A (yellow) was determined at 24 hpi through immunocytochemistry in presence of viral NS1 protein as a marker for infection. DNA counterstain is shown in blue. *P*-value ≤ 0.05 = *, *P*-value ≤ 0.01 = **, *P*-value ≤ 0.001 = ***, *P*-value ≤ 0.0001 = ****. Scale bar = 20 μm

We found that more than 75% of hiPSCs remained viable after exposure to 50 nM Rapamycin, an autophagy promoter, for 24 h (Fig. 4a), while Bafilomycin, which inhibits autophagic activity, was more toxic for these cells, as their treatment with the same concentration resulted in a 50% drop in cell viability (Fig. 4b). Infection followed by treatment with 50 nM Rapamycin and 5 nM Bafilomycin decreased the viability to below 40% within 24 h, which is slightly less than the effect of infection alone in the absence of these drugs (Fig. 4c, d, left panels). Although the viral titer at 24 h after treatment with autophagy inducer did not pass its initial titer at time-point zero, viral yield was increased ~tenfold by Rapamycin (Fig. 4c, right panel) but, conversely, was decreased ~tenfold in Bafilomycin-treated cells (Fig. 4d, right panel), suggesting that selected concentrations affect IAV replication in hiPSCs. The conversion of LC3β-I to LC3β-II, and the degradation of P62, were significantly upregulated in Rapamycin-treated cells in both infected and noninfected conditions, compared to mock or nontreated controls, whereas autophagy indicators in Bafilomycin-treated cells, either infected or noninfected, were expressed almost similar to mock and nontreated control (Fig. 4e), which confirms the efficacy of selected concentrations in altering viral-induced autophagy in hiPSCs. Both infected and noninfected Rapamycin-treated cells showed 60% or more loss in the expression of pluripotency markers, which is more substantial than the effect of infection without Rapamycin (Figs. 3 and 4f). In Bafilomycin-treated cells, pluripotency proteins did not change significantly, but infection with PR8 virus caused slight reduction in their expressions, which were still between twofold and fourfold greater than signals for Rapamycin-treated cells, showing that Bafilomycin reduces the negative effect of influenza-mediated autophagy on pluripotency. Moreover, although our western blotting indicated that Bafilomycin-treated cells can express IAV NS1 protein almost equal to infected nontreated cells, we found that florescence signals for NS1 production were severely decreased and clustered under the influence of Bafilomycin, while the NS1 expression was highly increased by Rapamycin in different assays, compared to normal nontreated infected cells (Figs. 3 and 4e, f). Since both pharmacological promotion of autophagy by Rapamycin and influenza upregulate autophagic activity by inhibiting mechanistic target of rapamycin (mTOR), these results suggest a relationship between influenza-induced autophagy and loss of pluripotency.

### PR8 infection alters the hiPSC proteome

**Fig. 5:**
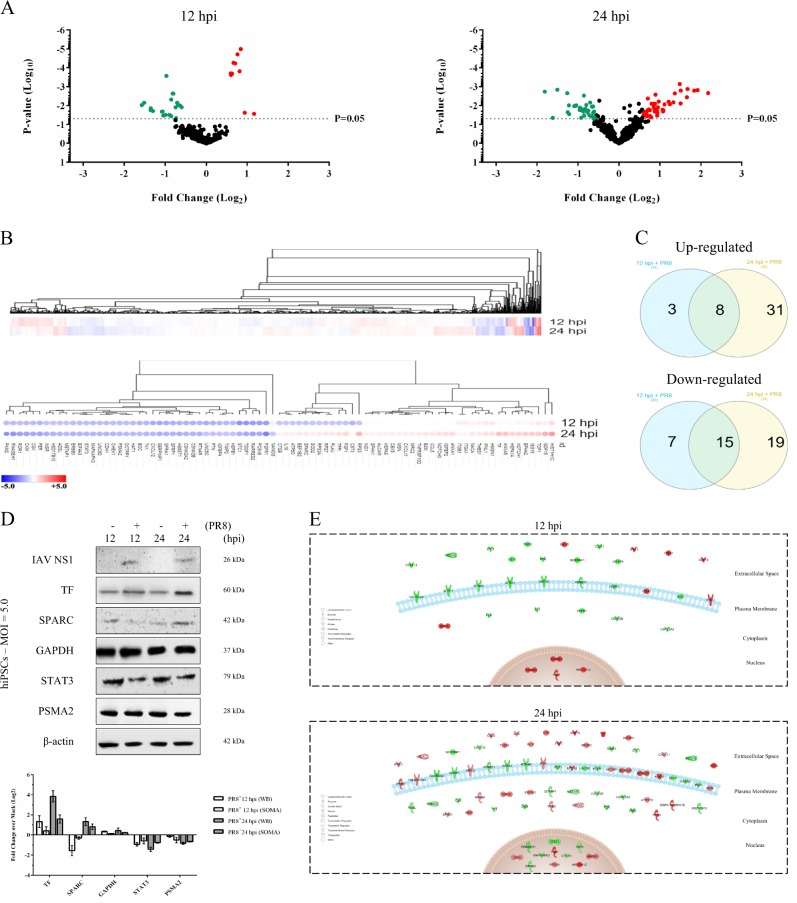
SOMAscan proteomic analysis of IAV-infected hiPSCs. **a** Volcano plots showing the expression fold changes and the significance of differentially expressed proteins at 12 and 24 hpi (*P*-value < 0.05). **b** The heatmap of the whole proteome (upper image) and the comparison (lower image) heatmap of differentially expressed proteins. **c** Venn diagram of the overlapping proteins between two time-points. Diagrams of significantly regulated proteins were plotted using InteractiVenn free online software. **d** Western blot confirmation of selected differentially regulated proteins from SOMAscan results. Western blot results are represented from multiple different gels. Various loading control band intensities were used from different gels to perform densitometry normalization and analysis. For validation, proteins were selected based on fold changes, availability of antibodies, and involvement in pluripotency and differentiation. These proteins include serotransferrin (TF), osteonectin (SPARC), glyceraldehyde 3-phosphate dehydrogenase (GAPDH), signal transducer and activator of transcription 3 (STAT3), and proteasome subunit alpha type-2 (PSMA2). Influenza NS1 also was probed to confirm successful infection. **e** The subcellular localizations of regulated proteins at 12 and 24 hpi. Graphical representation of cellular compartments was illustrated using pathway designer plugin in the IPA tool. Up- and downregulated proteins are highlighted in red and green, respectively

Our SOMAscan^®^ screening detected more significantly differentially expressed proteins at 24 hpi than at 12 hpi (Fig. 5a, Table 1). About 6% of the measured proteome underwent significant modifications by IAV (Fig. 5b). Most of upregulated and downregulated proteins at 12 hpi followed their significant altered pattern of expression to 24 hpi (Fig. 5c). Validating the differential regulations of some proteins revealed similar expression patterns (Fig. 5d). Most of the affected proteins at 12 hpi were mapped in extracellular (secretory) (39.3%) and plasma membrane (30.3%) regions. These two areas collectively contained 54.7% of influenza-modulated proteins at 24 hpi (Fig. 5e).

**Table 1:** hiPSC proteins affected by PR8 infection

		12 hpi		24 hpi	
Entrez gene symbol	Protein name	Fold change	*P*-value	Fold change	*P*-value
HIST1H1C	Histone cluster 1 H1 family member c	2.25	2.72E−02	4.52	2.16E−03
TOP1	DNA topoisomerase I	1.92	2.37E−02	2.83	7.57E−03
HMGN1	High mobility group nucleosome binding domain 1	1.80	1.00E−05	1.96	6.10E−03
IFNL1	Interferon lambda 1	1.76	1.51E−04	2.08	1.64E−02
ITGA1	Integrin subunit alpha 1	1.70	1.90E−05	1.47	5.45E−02
ITGB1	Integrin subunit beta 1	1.70	1.90E−05	1.47	5.45E−02
GDF15	Growth differentiation factor 15	1.64	5.70E−05	3.78	1.51E−03
THBS1	Thrombospondin 1	1.59	5.40E−05	1.84	8.02E−03
NANOG	Nanog homeobox	1.55	2.03E−04	−1.06	6.03E−01
NACA	Nascent polypeptide-associated complex alpha subunit	1.52	2.34E−04	1.90	1.83E−02
ISG15	ISG15 ubiquitin-like modifier	1.52	1.97E−04	2.84	1.58E−03
HSPB1	Heat shock protein family B (small) member 1	−1.51	1.20E−02	−1.10	4.54E−01
TIMP2	TIMP metallopeptidase inhibitor 2	−1.56	9.90E−03	−1.10	3.49E−01
BOC	BOC cell adhesion associated, oncogene regulated	−1.61	9.02E−03	−1.42	1.18E−01
IGFBP4	Insulin like growth factor binding protein 4	−1.63	6.99E−03	−1.14	2.80E−01
FER	FER tyrosine kinase	−1.66	4.61E−02	−2.38	2.23E−02
TK1	Thymidine kinase 1	−1.67	1.22E−02	−1.62	1.37E−02
RNASEH1	Ribonuclease H1	−1.68	1.00E−05	−2.83	1.42E−03
CST3	Cystatin C	−1.74	2.29E−03	1.20	5.34E−02
RTN4R	Reticulon 4 receptor	−1.76	2.26E−03	−1.84	1.56E−02
CSNK2B	Casein kinase 2 beta	−1.80	4.76E−03	−1.78	1.61E−02
CSNK2A2	Casein kinase 2 alpha 2	−1.80	4.76E−03	−1.78	1.61E−02
HS6ST1	Heparan sulfate 6-O-sulfotransferase 1	−1.81	3.62E−02	−1.91	1.73E−02
PTN	Pleiotrophin	−1.86	3.13E−02	−1.20	1.57E−01
SFRP1	Secreted frizzled related protein 1	−1.96	2.67E−04	−1.80	5.91E−03
UNC5C	UNC-5 netrin receptor C	−1.99	3.14E−02	−1.15	2.83E−01
GFRA1	GDNF family receptor alpha 1	−2.06	3.40E−02	−1.80	3.44E−02
CXCL12	C-X-C motif chemokine ligand 12	−2.08	2.03E−02	−1.54	2.21E−02
SERPINF1	Serpin family F member 1	−2.11	2.08E−02	−1.94	1.68E−02
PCSK9	Proprotein convertase subtilisin/kexin type 9	−2.46	1.93E−02	−2.07	9.92E−03
RARRES2	Retinoic acid receptor responder 2	−2.55	1.37E−02	−2.33	9.53E−03
FGFR1	Fibroblast growth factor receptor 1	−2.56	1.70E−02	−3.50	1.81E−03
TDGF1	Teratocarcinoma-derived growth factor 1	−2.85	6.99E−03	−2.12	1.05E−02
STC1	Stanniocalcin 1	−2.97	9.27E−03	−1.89	4.41E−02
RPS3	Ribosomal protein S3	−1.13	5.69E−01	3.58	1.58E−03
ANXA5	Annexin A5	1.14	3.55E−01	3.19	1.31E−03
TF	Transferrin	1.39	3.40E−01	3.16	3.58E−03
EPHA2	EPH receptor A2	1.39	1.40E−01	2.80	7.14E−04
NOTCH1	Notch 1	1.42	2.29E−01	2.58	2.26E−03
HSPA1A	Heat shock protein family A (Hsp70) member 1A	1.25	1.17E−01	2.52	6.64E−03
FGF1	Fibroblast growth factor 1	−1.28	3.95E−01	2.36	8.33E−03
ANXA1	Annexin A1	1.10	6.39E−01	2.32	6.23E−03
IGFBP2	Insulin like growth factor binding protein 2	1.00	9.94E−01	2.17	8.64E−03
TFPI	The tissue factor pathway inhibitor	−1.05	8.21E−01	2.12	1.76E−02
NOTCH3	Notch 3	1.19	4.36E−01	2.11	2.02E−02
CCL5	C-C motif chemokine ligand 5	1.24	2.87E−01	1.94	1.23E−02
PLAU	Plasminogen activator, urokinase	−1.00	9.80E−01	1.91	2.46E−02
RPS7	Ribosomal protein S7	−1.00	9.77E−01	1.91	3.39E−02
B2M	Beta-2-microglobulin	1.04	8.52E−01	1.86	2.61E−03
TNFRSF10D	TNF receptor superfamily member 10d	1.17	1.99E−01	1.84	2.06E−02
SPARC	Secreted protein acidic and cysteine rich	−1.23	2.02E−01	1.83	1.48E−02
RPS3A	Ribosomal protein S3A	−1.03	8.53E−01	1.81	8.07E−03
EEF1B2	Eukaryotic translation elongation factor 1 beta 2	−1.10	6.86E−01	1.80	1.19E−02
MRC2	Mannose receptor C type 2	1.15	5.09E−01	1.74	7.98E−03
CBX5	Chromobox 5	1.08	6.93E−01	1.73	4.16E−02
CXCL11	C-X-C motif chemokine ligand 11	1.11	6.10E−01	1.70	3.60E−02
SOD2	Superoxide dismutase 2	−1.08	5.51E−01	1.69	1.74E−02
MSN	Moesin	1.14	5.16E−01	1.66	1.57E−02
ATP5O	ATP synthase peripheral stalk subunit OSCP	−1.08	7.09E−01	1.63	8.31E−03
IL19	Interleukin 19	−1.05	7.26E−01	1.62	1.81E−02
ALCAM	Activated leukocyte cell adhesion molecule	1.29	2.87E−01	1.60	3.65E−02
HSPA8	Heat shock protein family A (Hsp70) member 8	1.07	5.76E−01	1.60	2.78E−02
SPHK1	Sphingosine kinase 1	1.17	5.26E−01	1.54	2.56E−02
NID1	Nidogen 1	1.25	3.90E−01	1.52	3.21E−02
CTSB	Cathepsin B	−1.01	9.41E−01	1.52	2.02E−02
HAT1	Histone acetyltransferase 1	−1.45	2.25E−01	−1.52	4.12E−02
DCTPP1	dCTP pyrophosphatase 1	−1.44	2.73E−01	−1.52	4.11E−02
METAP1	Methionyl aminopeptidase 1	−1.12	6.67E−01	−1.53	4.97E−02
PSMA2	Proteasome subunit alpha 2	−1.43	2.07E−01	−1.55	2.39E−02
CHEK1	Checkpoint kinase 1	−1.48	1.32E−01	−1.57	2.32E−02
CDH1	Cadherin 1	−1.39	2.16E−01	−1.58	2.24E−02
ADSL	Adenylosuccinate lyase	−1.19	2.60E−01	−1.58	3.77E−02
UNC5D	unc-5 netrin receptor D	−1.45	1.23E−01	−1.64	3.28E−02
MAPKAPK3	Mitogen-activated protein kinase-activated protein kinase 3	−1.41	2.15E−01	−1.68	1.84E−02
STAT3	Signal transducer and activator of transcription 3	−1.40	1.73E−01	−1.70	2.30E−02
EIF4A3	Eukaryotic translation initiation factor 4A3	−1.39	2.23E−01	−1.74	9.16E−03
ERBB3	erb-b2 receptor tyrosine kinase 3	−1.43	1.39E−01	−1.77	1.49E−02
HSD17B10	Hydroxysteroid 17-beta dehydrogenase 10	−1.11	6.21E−01	−1.82	2.06E−02
INSR	Insulin receptor	−1.38	2.73E−01	−1.93	1.28E−02
CSK	C-terminal Src kinase	−1.58	2.76E−01	−2.06	9.52E−03
CKM	Creatine kinase, M-type	−1.48	3.88E−01	−2.32	2.85E−02
ROR1	Receptor tyrosine kinase like orphan receptor 1	−1.35	2.60E−01	−2.39	2.18E−03
PKM2	Pyruvate kinase M1/2	−1.48	5.20E−01	−3.06	4.48E−02

### Influenza targets proteins controlling embryogenesis

**Fig. 6:**
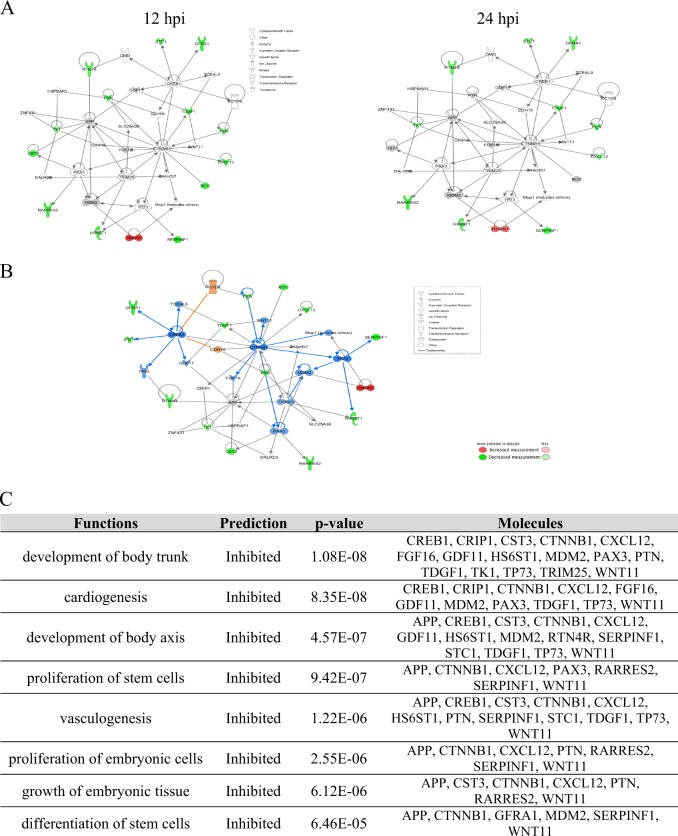
Influenza affects proteins regulating embryonic development network. **a** Changes in embryonic development network across two time-points. **b** IPA prediction on the expression patterns of molecules that belong to the embryonic development network but not found to be either differentially regulated or identified in our datasets. **c** Selection of some bio-functions that were predicted to be inhibited based on this network. These functions are mostly related to early differentiation phase of embryonic stem cells and embryogenesis. The datasets containing protein IDs, fold changes, and *P*-values were imported into the IPA software, and interacting networks were assembled for differentially expressed proteins. Up- and downregulated proteins are indicated in red and green, respectively; gray proteins denote that they were identified in this study but not affected; colorless proteins interact with various proteins in the network but were not recognized by our SOMAscan screening. IPA “Grow tool” and bioinformatic predictions were used to connect bio-functions to the network

Interconnecting networks of proteins were created using Ingenuity Pathways Analysis (IPA) software to determine interactions among dysregulated proteins in influenza-infected hiPSCs (Supplementary Fig. 1). Most of the significantly regulated proteins in the embryonic development network were downregulated by IAV (Fig. 6a). IPA also predicted potential inhibition or activation of other member molecules in this network that were not regulated or not found in our dataset (Fig. 6b). Experimental data and bioinformatic prediction connected this network to bio-functions related to stem cells and embryogenesis (Fig. 6c). These influenza-induced dysregulations in protein–protein interaction networks may negatively affect differentiation and embryogenesis.

### PR8 virus dysregulates pathways and bio-functions regulating cell differentiation

**Fig. 7:**
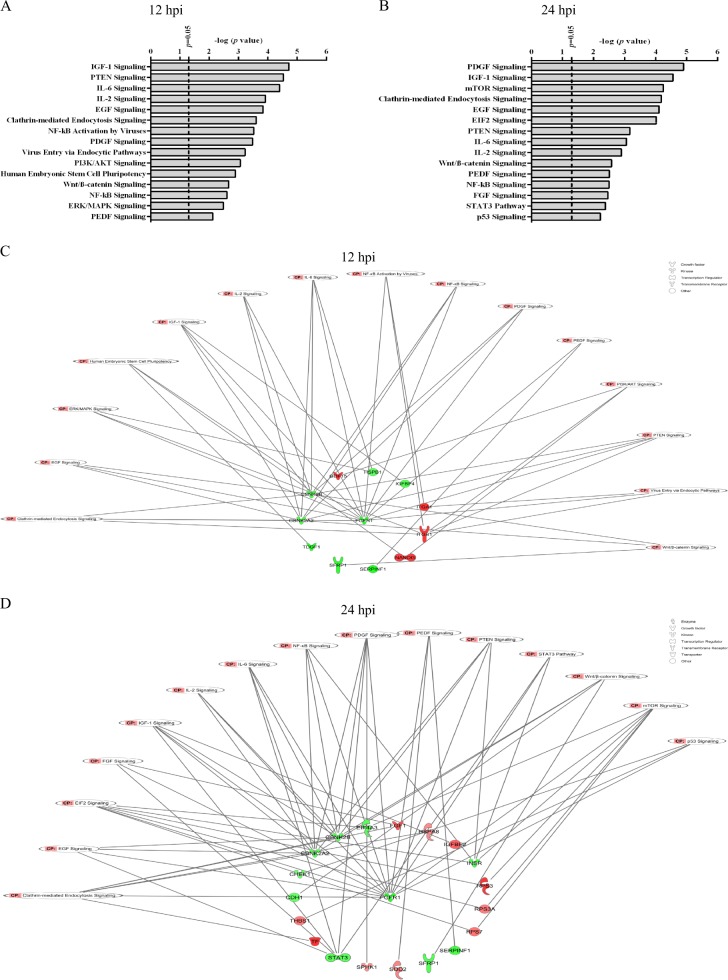
Top-affected canonical pathways in hiPSCs after PR8 infection. Protein IDs together with their *P*-values and levels of regulation were uploaded into IPA tool. In the canonical pathway section, *P*-values of pathways were automatically calculated by the software and top significantly affected pathways were graphed for 12 hpi (**a**) and 24 hpi (**b**). Because the affected member molecules from these targeted pathways were fewer than the total numbers of molecules present in those pathways based on SOMAscan screening, IPA software did not predict significant inhibition or activation patterns for the vast majority of influenza-modulated signaling pathways. **c**, **d** Network of common molecules mediating influenza-induced alterations into top-affected canonical pathways at 12 and 24 hpi. Up- and downregulated proteins are shown in red and green, respectively

**Fig. 8:**
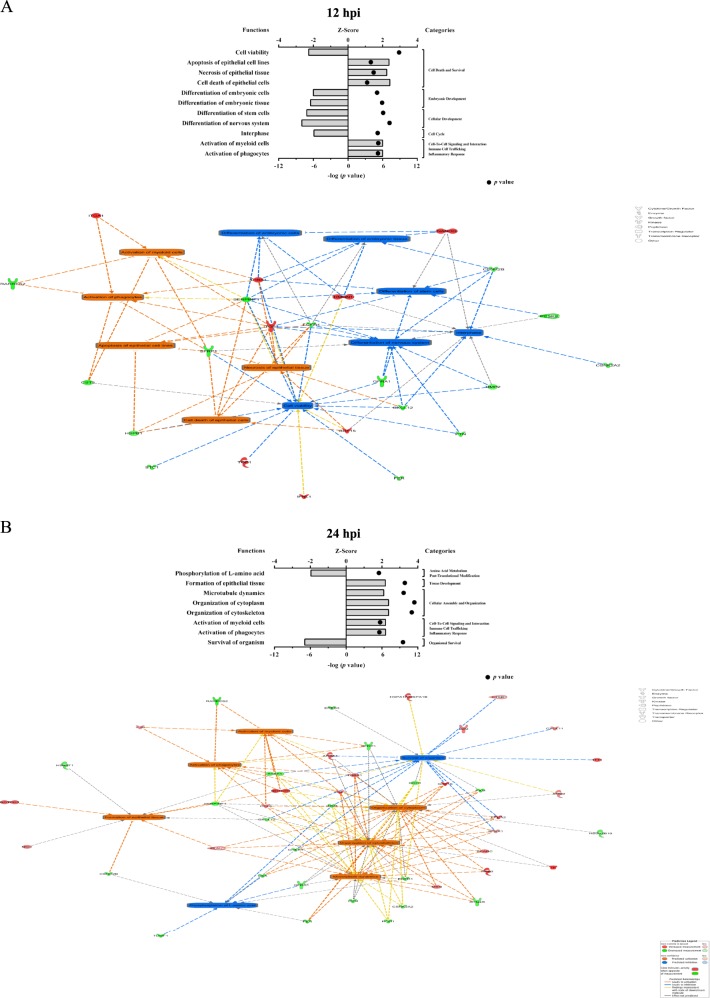
IPA prediction of top affected bio-functions by differentially expressed proteins at 12 hpi (**a**) and 24 hpi (**b**). In the bio-function section of IPA, activation and inhibition *z*-scores were automatically calculated by the software and top significantly affected functions were merged and displayed as networks with auto layout. *Z*-scores values of ≥1.96σ and ≤−1.96σ were specified as the criteria for activation and inhibition patterns

IPA identified IAV-modulated pathways in hiPSCs (Fig. 7a, b), some of which are involved in differentiation and pluripotency. The effects of IAV on the member molecules of these pathways are shown in Supplementary Fig. 2. We also generated networks of molecules altered by the virus that individually or collectively mediate modifications in affected pathways (Fig. 7c, d). IPA also predicted decreases in differentiation, embryogenesis, and cell viability, and increase in cell death by 12 hpi (Fig. 8a), indicating that IAV initially causes cell death and suppressed differentiation. Nevertheless, activation of cellular development at 24 hpi (Fig. 8b) suggests the possibility of differentiation.

## Discussion

Many questions concerning the effect of viruses on the unique characteristics of PSCs, like pluripotency, differentiation, and cell death signals, have remained unanswered. Here, we showed that IAV propagates restrictedly in hiPSCs. Wash et al. observed similar limited IAV replication in mESCs^14^. In our study, higher MOI did not promote PR8 replication but allowed synthesis of viral proteins, including NS1, which alone confirms the transport of viral ribonucleoproteins (vRNPs) into the nucleus, and initiation of transcription and translation. Compared to another (−)ssRNA viruses such as measles, which replicates more in iPSCs^23^, the assembly of progeny IAV viruses was disrupted in our experiments, even though viral proteins synthesized at higher MOI confirm the cell's transcription machinery is hijacked by IAV. It has been speculated that underdeveloped glycosylation in PSCs hinders viral haemagglutinin (HA) and neuraminidase (NA) maturation^14,24–27^, thereby resulting in not only reduced binding to cell receptors and less fusion with endosomal membrane but also decreased budding of new virions. The rapid proliferation of PSCs might also have negatively influenced the initial MOI and ultimately the ability of IAV to release measurable progeny, as already reported in ESCs infected with other (−)ssRNA viruses like La Crosse virus (LACV) and Sendai virus^28^. Growth conditions of these cells can also make barriers in simulating a normal in vivo infection. For instance, since iPSCs are grown in colonies on Matrigel-coated surfaces and cannot make confluent monolayers, viruses, as charged particles, might be limited in movement toward colonies because of being partially trapped in Matrigel^23,29^, suggesting efficacy of higher MOIs for sufficient attachment and entry.

We observed that PR8-infected hiPSCs develop CPE. This phenomenon was consistent with low cell viability. Similar CPE and loss in cell viability were reported in IAV-infected mESCs^14^. Some (−)ssRNA viruses like Measles with efficient replication and LACV with low viral yield, can also develop diverse types of CPE in hiPSCs and mESCs, respectively^23,28,30^. Our observation may suggest that if IAV infection occurs at the blastocyst stage, embryogenesis could be disrupted in cases of transplacental passage as the ICM is directly affected. We also attempted to discover the type of cell death that was elicited by IAV in hiPSCs with primary emphasis on apoptosis, the most common type of programmed cell death that can be induced by IAV through intrinsic and extrinsic mechanisms^31,32^. We showed that PR8 virus does not contribute to intrinsic apoptosis in hiPSCs, as noted by inactivation of caspases, but induces this type of cell death in lung epithelial A549 cells based on differential expression of caspases and apoptosis regulators. Such a cell-specific difference confirms unique expression profiles of intrinsic apoptosis executioners and regulators in hiPSCs. In somatic cells, apoptosis can be temporarily blocked by IAV NS1 protein at preliminary stages of infection via PI3k-Akt signaling pathway for expanding viral replication^33–35^. This early inhibition of apoptosis then is accompanied by apoptotic or necrotic pathways at subsequent phases of infection to promote cell death in infected cells and viral budding^36^. In our study, intrinsic apoptotic response might become activated later (e.g., after 24 hpi), which we did not investigate here because of decreased cell viability. IAV-induced cell death in hiPSCs might also have occurred via caspase-independent mechanisms (e.g., by extrinsic apoptosis or alternative cell death pathways).

The acidification of endosome-lysosome system is needed for proper function of lysosomal enzyme and fusion of autophagosome with lysosome to complete autophagy, the pathway controlling degradation of dysfunctional organelles and recycling of cellular components^37^. This also provides an optimal condition for uncoating IAV particles and release of RNPs^36,38,39^. Interestingly, IAV induces autophagy in a variety of mammalian cells by inhibiting mTOR^17,40,41^. Considering the possibility of cell death with autophagy^42^, we detected activation of autophagy in both influenza-infected A549s and hiPSCs. To our knowledge, this is the first evidence of virus-induced autophagy in PSCs, despite the lack of proof to directly link this activated mechanism to demonstrated cell death. After fertilization, autophagy levels were immediately upregulated in murine oocytes^43^. Subsequently, autophagy is inhibited between the one- and two-cell stage but can be increased again at the four-cell stage^44^. However, this mechanism is not clearly understood under pluripotent state and seemed to be stable and dispensable in ESCs. Consequences of suppressed autophagy in PSCs can be evolved during embryogenesis or later in differentiated cells and include neuronal inclusions, reduced adipose mass, unwanted accumulation of damaged organelles, and defective embryoid body formation in culture with insufficient ATP production^45,46^. In contrast, implications of excessive abnormally induced autophagy may manifest subtlety in undifferentiated PSCs or in derived tissues and yet remain to be determined. The ubiquitin-proteasome system (UPS) may compensate for intrinsically low levels of autophagy in pluripotent state, due to high proteasome activity in these undifferentiated cells. Conversely, polyubiquitinated proteins are highly expressed in differentiated cells, and proteasome activity is progressively inhibited during differentiation process^47^. Furthermore, higher levels of autophagy were naturally observed during the differentiation of ESCs, probably through degrading protein complexes regulating pluripotency^48^. Many viruses develop mechanisms to evade, subvert, or exploit cellular processes for their benefit. We speculate that IAV induces autophagy in hiPSCs differently from its normal upregulation upon certain conditions. Such an altered viral-triggered mechanism under pluripotency state suggests the possibility of abnormal differentiation, as elevated levels of autophagy are usually seen in differentiating PSCs. It is thus necessary to evaluate the pluripotency and capacities of these cells for normal differentiation into germ layers after IAV infection to decipher the role of influenza-induced autophagy in abnormal embryonic specification. Hypothetically, with regards to demonstrated cell death, the virus may simultaneously put iPSCs under differentiation and promote autophagy, or may keep them in an undifferentiated state while increasing autophagic activity. It is also possible that the expected differentiation is not directly modulated by the virus itself and could be the outcome of virus-induced excessive autophagy. In all cases, cell death could be executed via different pathways and whether autophagy is actually playing a role in the cellular demise of iPSCs remains unclear^42^.

The effects of viral replication or entry on pluripotency have not yet been described for most viruses. Moreover, specifying the level of pluripotency indicates the possibility of differentiation in virus-infected iPSCs. We found influenza-infected hiPSCs to exhibit a remarkable decrease in the expression of pluripotency regulating proteins Nanog, Sox2, and Oct-4A. This is the first evidence so far that proves PR8 infection negatively affects the pluripotency of hiPSCs. Measles, as the only other (−)ssRNA virus tested for its effect on pluripotency, was incapable of altering this intrinsic feature of PSCs, but blocks their directed differentiation to germ layers due to extensive CPEs^23^. Significant levels of cell death that happened by 24 hpi and necessity of using higher MOI did not allow us to test the capability of PR8-infected cells for further differentiation into germ layers. Nevertheless, reduced pluripotency noted in the current study together with our findings regarding IAV-induced autophagy highlight a link between excessive autophagy and loss of pluripotency. Some studies suggest that mTOR acts as the regulator of pluripotency and self-renewal in hESCs, in addition to its central role in the regulation of the autophagy pathway and triggering several downstream pathways including PI3-kinase/Akt and AMP-activated protein kinase^44,49,50^. Zhou and coworkers reported a set of outcomes in hESCs after inhibition or depletion of mTOR, including considerable reduction in expression of Oct4 and Sox2, elevated mesoderm and endoderm differentiation, and limited proliferation^51^. At 24 hpi, we obtained similar results; decreased expression of Oct4 and Sox2 after influenza-induced autophagy. Additionally, our SOMAscan proteomic screening revealed evidence of potentially abnormal differentiation based on dysregulated proteins at the same postinfection time point. In A549 cells, mTOR inhibition with Rapamycin was reported to upregulate autophagy and production of viral NP and NS1 proteins, but did not increase viral yield^36^. We also pharmacologically inhibited mTOR in hiPSCs using Rapamycin and noticed significant loss of pluripotency, but higher levels of viral growth, viral protein production, and autophagic activity. In our study, PR8-infected hiPSCs showed not only evidence of viral protein production but also restricted viral replication and caspase-independent or nonapoptotic cell death under excessive autophagic activity. Irrespective of our previous assumptions about the type of cell death and possible reasons for limited viral replication, it has also been hypothesized that mTOR suppression stimulates upstream receptor tyrosine kinase signaling, subsequently activating the Akt/PKB pathway, which may inhibit apoptosis. According to a transcriptomic analysis, mTOR is upregulated during differentiation and afterward compared to its expression under the pluripotent state^52^. We did not directly evaluate the expression of mTOR in IAV-infected hiPSCs. However, our SOMAscan proteomic screen showed that some mTOR-signaling molecules like FGFR1 and INSR1, that are expected to be upregulated upon pathway activation, were downregulated by 24 hpi, suggesting potential IAV-triggered inhibition of this pathway. Hence, our results, and those of others, suggest that PR8 virus promotes autophagy in these PSCs, possibly by downregulating mTOR, which decreases pluripotency, ultimately inducing abnormal differentiation. Further investigations are required to explain the initiation of influenza-induced autophagy in PSCs and its possible effect on differentiation.

Our proteomic analyses showed that PR8 virus altered the proteome of hiPSCs. This observation is consistent with what has been previously reported about the limited responses of these cells to most viruses^53^. Likewise, mESCs had also indicated minor dysregulations at the transcriptomic level after 6 h of infection with influenza^14^. Such limited host responses to influenza infection might be because of inhibited viral replication in these pluripotent cell populations. At 12 hpi, although a few proteins like Interleukin-29 (IL-29/IFNL1) and Interferon-stimulated gene 15 (ISG15), that are involved in type 3 and 1 interferon responses, were upregulated less than twofold, an apparent deficiency was noticed in the expression of proteins related to innate immune responses against viruses. Inactivated immune responses were already seen in PSCs after different viral or bacterial infections and are mainly due to underdeveloped innate immunity and mutual inhibition between the IFN system and pluripotent state in these cells^53–55^. Highly conserved regulation of transcription in ESCs can be considered another condition that may downregulate interferon-stimulated genes for maintaining pluripotency^14,56^. Bioinformatic analyses of differentially regulated proteins at 12 hpi also highlighted significant negative impacts on activation of some canonical pathways controlling immune responses, including leukocyte extravasation signaling, IL-2 signaling, and IL-6 signaling. It is possible that the 1.5-fold upregulation of ISG15 at this time point can be partially explained by the intrinsic immature immunity of PSCs to IAV, as it has been shown that this protein can ISGylate IAV NS1 protein which leads to the inefficiency of NS1 and limited viral replication, the same situation we observed experimentally. IPA software also predicted some potentially affected bio-functions, and these data collectively support the induction of cell death by either necrosis or apoptosis, reduction of cell viability, and inhibition of differentiation in ESCs. We found these bioinformatic predictions reliable and consistent with what we have already acquired on induction of cell death and stable pluripotency at 12 hpi. Even though we did not show the normal expression of pluripotency markers by 12 hpi through other assays, our proteomic screening determined the upregulation of Nanog, thus confirming the pluripotency of PR8-infected hiPSCs and repressed differentiation at the mentioned time point. In contrast, at 24 hpi, IPA detected unexpected dysregulations among some top-affected canonical pathways that are involved in processing differentiation and embryogenesis. More studies should be carried out to test whether IAV-induced changes could influence the normal differentiation of PSCs into germ layers.

In conclusion, our results indicated IAV causes cytopathology in hiPSCs and can reduce their viability and pluripotency. It was also noticed that such an IAV-triggered decline in pluripotency is associated with an excessive level of autophagy. Moreover, our proteomic screening suggests dysregulations in several cellular pathways and bio-functions regulating differentiation. Further studies are needed to discover the molecular mechanisms involved in initiation of influenza-induced autophagy in PSCs and their possible effects on the signaling pathways controlling embryogenesis.

## Materials and methods

### Cells and viruses

hiPS cells were generated from human peripheral blood mononuclear cells (PBMCs) using the Sendai virus Kit (Life Technologies), as described previously^57^. All protocols were approved by the Research Ethics Board (REB) of the University of Manitoba. Human influenza virus strain A/PR/8/34 (H1N1; PR8), an attenuated mouse-adapted strain, was amplified in MDCK cells by infecting at MOI of 0.01 for 48 h and concentrated at 64,000 × *g* for 2 h at 4 °C. The virus was then titered by the plaque assay on MDCK cells.

### Infection and plaque assay

After washing semiconfluent hiPSC colonies 2× with 1× phosphate buffered saline (PBS; 137 mM NaCl, 0.3 mM KCl, 0.8 mM Na_2_HPO_4_, 0.1 mM KH_2_PO_4_), cells were infected with PR8 virus diluted in E8 medium to achieve different MOIs, including 0.1, 1, and 5 plaque forming units (PFU)/cell. To compare IAV growth kinetics in hiPSCs with other influenza-permissive cell lines, A549 and MDCK cells also were infected at the same MOIs by diluting the PR8 virus in gel saline (137 mM NaCl, 0.2 mM CaCl2, 0.8 mM MgCl2, 19 mM HBO3, 0.1 mM Na2B4O7, 0.3% (w/v) gelatin). An equivalent number of cells were mock-infected using either only E8 medium for PSCs or gel saline for other cells. At 12 and 24 hpi, infected and mock-infected hiPS and A549 cells were harvested for immunoblotting. To quantify the virus yield by the plaque assay, supernatants were collected from all three cell types at assigned time points and serially diluted 1:10 in gel saline. Diluted supernatants then were added to subconfluent monolayers of MDCK cells plated in six-well dishes. Following an hour adsorption, cells were overlaid with 0.8% Avicel in FBS-free 1× DMEM media containing 2 mM l-glutamine, 2 mM sodium pyruvate, and 1× MEM nonessential amino acids, and supplemented with 2.5 μg/mL trypsin, 1× gentamicin and 1× amphotericin B. After 72 h incubation at 35 °C to permit plaque formation, cells were fixed with 2% formaldehyde for 30 min and then stained with crystal violet for 1 h. Viral titer was calculated as PFU/mL by counting plaques 4 h after washing stained cell monolayers^58^.

### Immunoblotting

At time points 12 and 24 hpi, mock- and influenza-infected hiPS and A549 cells were scraped into cold PBS, then pelleted at 500 × *g* for 6 min, and lysed for 15 min in mammalian protein extraction reagent (M-PER^TM^, Thermo Scientific) supplemented with HALT^TM^ protease inhibitor (Thermo Scientific). After clearing cell lysates by centrifugation at 14,000 × *g* for 15 min, supernatant protein contents were collected, and the BCA Protein Assay Kit (Pierce, Thermo Scientific) was applied to measure protein concentrations. Equal amounts of proteins were loaded per lane into SDS-polyacrylamide gels (SDS-PAGE), fractionated, and transferred to Immobilon-P polyvinylidene difluoride membranes (Millipore). Membranes were blocked with 5% skim milk in Tris-Buffered Saline buffer containing 0.1% Tween 20 for 2 h, and then incubated overnight with the desired primary antibodies at 4 °C. Influenza primary anti-NP, -M1, and -NS1 antibodies were developed in-house^59^. Primary antibodies for caspases-3, -7, -9, P53, Nanog, Sox2, Oct-4A, Bax, Bcl-2, PSMA2, STAT3, SPARC, GAPDH, p62, and β-actin were purchased from Cell Signaling Technology. The LC3β and Atg5 antibodies were obtained from Invitrogen^TM^ and anti-Transferrin antibody was purchased from Abcam. Following overnight incubation with primary antibodies, membranes were probed with either rabbit or mouse HRP-conjugated secondary antibody (Cell Signaling) for 1 h at room temperature, and the bands were visualized through enhanced chemiluminescence detection machine (Amersham-Pharmacia Biotech). ImageJ software was used to quantify virus-to-mock ratios from the intensity of visualized bands. Blot quality was optimized for contrast and brightness using image settings plugin of Microsoft Word.

### Analysis of cellular morphology

To examine PR8-induced CPE development, infected and mock-infected hiPSCs were assessed by inverted microscopy (Nikon TE-2000) at intended MOIs and photographed using a Canon A700 camera. The analysis of stem cell colony mass and size was done through crystal violet staining in a 12-well plate. After washing three times with PBS, hiPSCs were fixed with 4% paraformaldehyde for 15 min and then stained with 0.5% crystal violet solution in 4% paraformaldehyde for 10 min. The stained plate was washed twice with water, and infected colonies were evaluated and compared to mock-infected wells on the next day.

### Assessment of cell viability

The trypan blue exclusion assay was used to determine cell viability. Briefly, PR8-infected or mock-infected hiPSCs were harvested at various postinfection time points by brief trypsinization to break up the colonies. Single cells derived from disaggregated colonies then were washed, stained with trypan blue solution and placed on a hemocytometer for counting. The percentage of viable cells was calculated based on the number of dead (blue) cells divided by the total number of cells counted, multiplied by 100. The cytotoxicity of autophagy drugs (Rapamycin and Bafilomycin) was determined by the WST-1 cell proliferation assay (Roche, Germany) according to the manufacturer's instructions. In brief, hiPSCs or A549s grown in 96‐well plates were treated with WST‐1 reagent for 90 min at 37 °C. Colorimetric changes were measured, and cell viability then was calculated compared with controls.

### Indirect IF microscopy

For IF staining, iPSCs were grown to 60% confluence on chamber slides and then infected or mock-infected with the PR8 virus at MOI = 5. After fixing with 4% paraformaldehyde for 15 min at 12 and 24 hpi, cells were permeabilized with 0.1% Triton X-100 in PBS for 5 min and blocked with 3% BSA blocking solution for 90 min. Afterward, cells were incubated overnight 4 °C with primary anti-Nanog, -Sox2, -Oct-4A (all three from Cell Signaling), -NS1, -NP, and -M1 (all three made in-house) antibodies diluted in 1% BSA and PBS. After overnight incubation, cells were washed 5× with PBT buffer (PBS with 0.2% Tween-20) and treated for 1 h with Alexa Fluor™ 488 and 546 (Invitrogen) secondary antibodies. Slides were then mounted using DAPI-Prolong^®^ Gold Antifade for nuclear staining, sealed and preserved at 4 °C for imaging on the following day. Fluorescent images were acquired at 20× and 40× objective by Zeiss Axio Observer Z1 inverted microscope and further optimized with AxioVision 4.8.2 software. ImageJ software was used to quantify the levels of florescence signal intensities.

### Inhibition and activation of autophagy

To establish a relationship between autophagy induction and loss of pluripotency, autophagic activity was blocked using autophagy inhibitor Bafilomycin A1 (Cayman Chemical), which inhibits autophagy through both hindering vacuolar acidification required for autophagosome maturation and mTOR activation^60,61^. Rapamycin (Sigma-Aldrich), an mTOR inhibitor and autophagy inducer^62^, was utilized for elevating the level of autophagy. Both drugs were used in four different concentrations of 4000, 500, 50, and 5 nM for cytotoxicity analyses. Based on cell viability results, lower concentrations were selected for evaluating viral growth, autophagy, and pluripotency. In influenza-infected conditions, cells were first adsorbed with the virus at MOI of 5.0 for 1 h and then treated with selected concentration of Bafilomycin (5 nM) or Rapamycin (50 nM) mixed in cell-specific culture medium.

### SOMAscan screening

After measuring protein concentrations in three biological replicates by the BCA assay, cell lysates were adjusted to 200 μg/ml and sent for proteomic screening using an in-house SOMAscan assay platform (version 1.3), a novel multiplexed system that uses modified nucleotides with high affinity known as SOMAmers (Slow Off-rate Modified Aptamers) to evaluate changes in the expression of intracellular proteins related to various biological processes^63^. In total, the expressions of 1307 proteins were determined from lysates isolated at 12 and 24 hpi from mock-infected and infected hiPSCs at MOI of 5. Raw specific protein abundance values were obtained as relative fluorescence units (RFU). To sort out modified proteins, we calculated *z*-scores separately for each biological replicate to normalize all screened proteins at a 95% confidence level cutoff. *Z*-scores values of ≥1.96σ and ≤−1.96σ were specified as the criteria for up- and downregulation patterns.

### Statistical and bioinformatic analysis

Data were collected in triplicate from all experiments. For growth curves, cell viability, and band intensity analyses, statistical differences were assessed by one-way or two-way ANOVA, graphed using Graph Pad Prism 6.0, and results were reported as means ± SDE. *P*-values < 0.05 were considered statistically significant. To analyze SOMAscan results, RFU raw expression values were imported into Microsoft Excel and converted to Log_2_ for calculating fold changes. *P*-values were determined using the two-tailed Student's t-test. To compare various biological replicates, *z*-scores were calculated from protein ratios within each replicate, as previously described^58,63^. Briefly, all fold changes not deemed to be significant by *t*-test were examined by *z*-score, expressing each value as its number of standard deviations away from the population mean. Protein IDs from the whole examined proteome were uploaded to and analyzed by IPA software. Panther databases were utilized to classify all significantly expressed proteins in gene ontology categories. Venn diagrams and heatmap were plotted using InteractiVenn and MORPHEUS (developed by Broad Institute, Cambridge, MA, USA) free online software, respectively.

## Supplementary information

Supplementary Figure 1

Supplementary Figure 2

Supplementary figure legends
