# Supplementary material for: Influenza a virus-triggered autophagy decreases the pluripotency of human-induced pluripotent stem cells
Source: Cell Death Dis. 2019 Apr 18;10(5):337. doi: 10.1038/s41419-019-1567-4 (PMC6472374; doi:10.1038/s41419-019-1567-4)
Supplement: Supplementary file 2 — Supplementary Figure 2 [file 41419_2019_1567_MOESM2_ESM.docx]

| IGF-1 signaling   | PTEN signaling   | IL-6 signaling   |
| --- | --- | --- |
| IL-2 signaling   | EGF signaling   | Clathrin-mediated endocytosis signaling   |
| PDGF signaling   | Wnt/β-catenin signaling  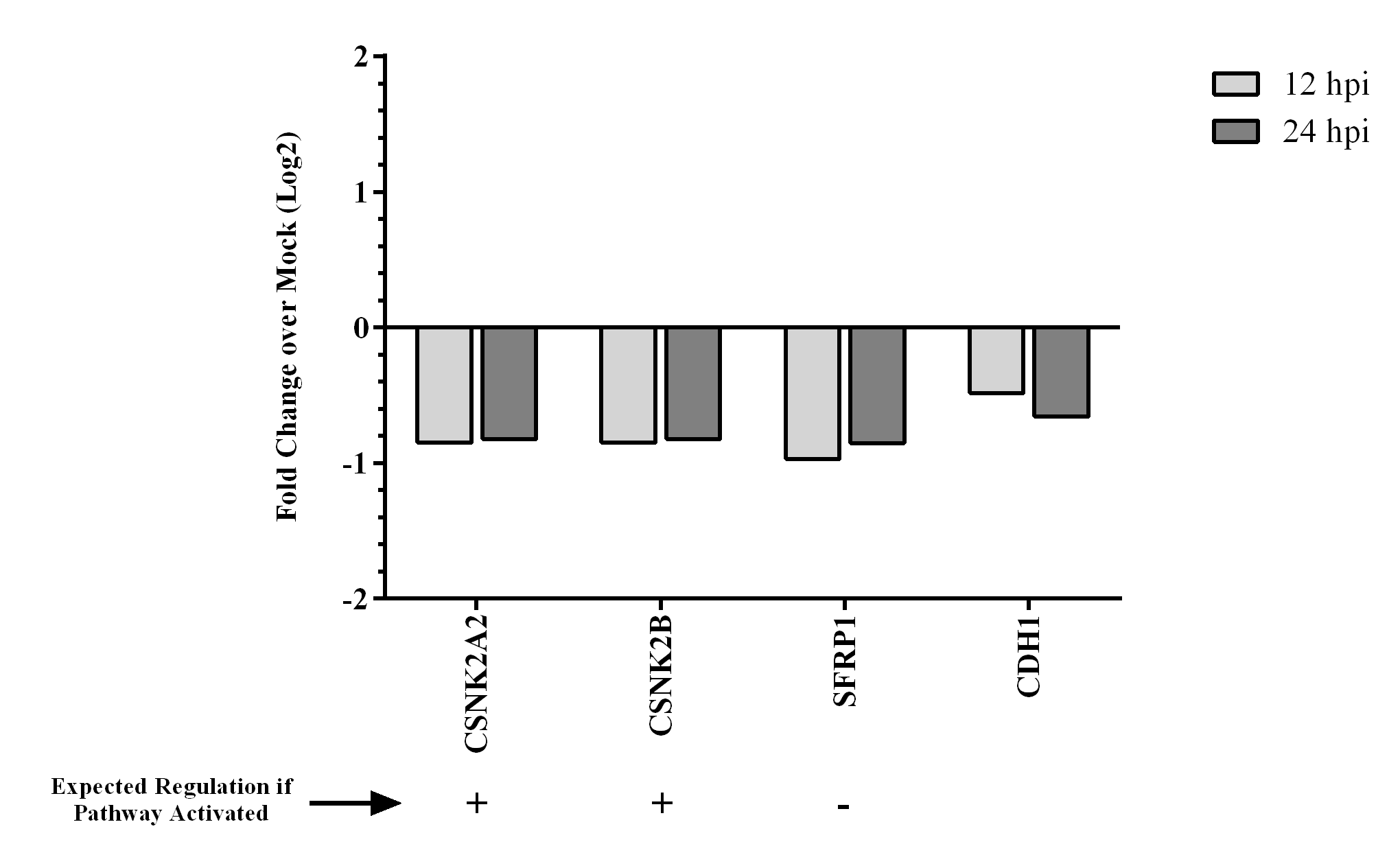 | NF-κB signaling   |
| PEDF signaling   | NF-κB activation by viruses   | Virus entry via endocytic pathways   |
| PI3K/AKT signaling   | Human embryonic stem cell pluripotency   | ERK/MAPK signaling   |
| mTOR signaling   | EIF2 signaling   | FGF signaling   |
| STAT3 pathway   | p53 signaling   |  |
