# Supplementary material for: Influenza a virus-triggered autophagy decreases the pluripotency of human-induced pluripotent stem cells
Source: Cell Death Dis. 2019 Apr 18;10(5):337. doi: 10.1038/s41419-019-1567-4 (PMC6472374; doi:10.1038/s41419-019-1567-4)
Supplement: Supplementary file 3 — Supplementary figure legends [file 41419_2019_1567_MOESM3_ESM.docx]

**Supplementary Figure Legends**

**Supplementary Figure 1. Molecular networks of regulated proteins.** IPA software-generated interacting networks based on differentially regulated proteins at 12 hpi (A) and 24 hpi (B). Interconnecting networks were created based on direct relationships by uploading datasets containing protein IDs, fold changes compared to mock-infected cells and p-values into the IPA program.

**Supplementary Figure 2. Impact of IAV on the regulation patterns of member molecules from top affected canonical pathways.** IAV-modulated modifications in member molecules of various pathways were characterized by IPA based on the former reports on the expected regulation patterns in affected molecules upon activation of a pathway under normal conditions.
