# Supplementary material for: Influenza a virus-triggered autophagy decreases the pluripotency of human-induced pluripotent stem cells
Source: Cell Death Dis. 2019 Apr 18;10(5):337. doi: 10.1038/s41419-019-1567-4 (PMC6472374; doi:10.1038/s41419-019-1567-4)
Supplement: Supplementary file 1 — Supplementary Figure 1 [file 41419_2019_1567_MOESM1_ESM.docx]

A

| **12 hpi** | |
| --- | --- |
| Network 1  Cellular Development, Cellular Growth and Proliferation, Organismal Development   | Network 2  Embryonic Development, Organismal Development, Cellular Development   |
|  |  |

B

| **24 hpi** | |
| --- | --- |
| Network 1  Cell Death and Survival, Cell Cycle   | Network 2  Cellular Movement, Organismal Injury and Abnormalities   |
| Network 3  Cell Death and Survival, Cell-To-Cell Signaling and Interaction   | Network 4  Free Radical Scavenging, Small Molecule Biochemistry, Lipid Metabolism   |
